# Association of chronic kidney disease and end-stage renal disease with procedural complications and in-hospital outcomes from left atrial appendage occlusion device implantation in patients with atrial fibrillation: Insights from the national inpatient sample of 36,065 procedures

**DOI:** 10.1016/j.hroo.2021.08.002

**Published:** 2021-08-21

**Authors:** Muhammad Bilal Munir, Muhammad Zia Khan, Douglas Darden, Marin Nishimura, Sai Vanam, Deepak Kumar Pasupula, Zain Ul Abideen Asad, Abhishek Bhagat, Salman Zahid, Mohammed Osman, Sudarshan Balla, Frederick T. Han, Ryan Reeves, Jonathan C. Hsu

**Affiliations:** ∗ Section of Electrophysiology, Division of Cardiology, University of California San Diego, La Jolla, California; † Division of Cardiovascular Medicine, West Virginia University Heart & Vascular Institute, Morgantown, West Virginia; ‡ Division of Cardiology, MercyOne North Iowa Medical Center, Mason City, Iowa; § Division of Cardiology, University of Oklahoma Health Sciences Center, Oklahoma City, Oklahoma; ‖ Division of Cardiology, University of Arizona College of Medicine, Phoenix, Arizona; ¶ Department of Medicine, Rochester General Hospital, Rochester, New York

**Keywords:** Chronic kidney disease, End-stage renal failure, Left atrial appendage occlusion, Outcomes, Watchman

## Abstract

**Background:**

Left atrial appendage occlusion (LAAO) has emerged as an alternative strategy to oral anticoagulation for mitigating ischemic stroke risk in selected patients with atrial fibrillation (AF), but safety data in patients with significant kidney disease are limited.

**Objective:**

To determine the association of chronic kidney disease (CKD) and end-stage renal disease (ESRD) with procedural complications and in-hospital outcomes after LAAO in AF patients.

**Methods:**

Data were extracted from National Inpatient Sample for calendar years 2015–2018. Watchman implantations were identified on the basis of International Classification of Diseases, 9th and 10th Revision, Clinical Modification codes of 37.90 and 02L73DK. The outcomes assessed in our study included complications, inpatient mortality, and resource utilization with LAAO.

**Results:**

A total of 36,065 Watchman recipients were included in the final analysis. CKD (9.8%, n = 3545) and ESRD (3%, n = 1155) were associated with a higher prevalence of major complications and mortality in crude analysis compared to no CKD. After multivariate adjustment for potential confounders, CKD was associated with length of stay (LOS) >1 day (adjusted odds ratio [aOR] 1.355; 95% confidence interval [CI] 1.234–1.488), median cost >$24,663 (aOR 1.267; 95% CI 1.176–1.365), and acute kidney injury (aOR 4.134; 95% CI 3.536–4.833), while ESRD was associated with in-patient mortality (aOR 7.156; 95% CI 3.294–15.544).

**Conclusion:**

The prevalence of CKD and ESRD was approximately 13% in AF patients undergoing Watchman LAAO implantations. CKD was independently associated with prolonged LOS, higher hospitalization costs, and acute kidney injury, while ESRD was independently associated with in-patient mortality.

## Graphical abstract

**Figure:**
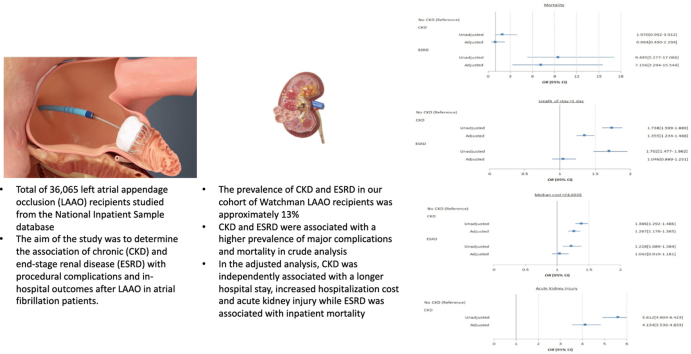

**Key Findings**

▪ Anticoagulation strategy is clinically challenging in atrial fibrillation patients with significant renal disease, as these patients have heightened stroke and bleeding risk. More recently, left atrial appendage occlusion has emerged as an alternative strategy to oral anticoagulation in selected patients with atrial fibrillation.
▪ In this largest national registry of atrial fibrillation patients undergoing left atrial appendage occlusion, the prevalence of significant renal disease (chronic kidney disease and end-stage renal disease) was approximately 13%.
▪ Chronic kidney disease was associated with increased length of stay, higher hospitalization costs, and acute kidney injury while end-stage renal disease was associated with inpatient mortality in our cohort of atrial fibrillation patients undergoing left atrial appendage occlusion based on adjusted analyses.

## Introduction

Atrial fibrillation (AF) is the most common cardiac arrhythmia encountered in clinical practice.^1^^,^^2^ The most significant adverse event associated with AF is stroke, and strokes related to AF are generally more disabling when compared to strokes not associated with AF.^3^^,^^4^ Chronic kidney disease (CKD) and end-stage renal failure (ESRD) are common comorbidities encountered in patients with AF.^5^, ^6^, ^7^ The management of oral anticoagulation in AF patients with CKD and ESRD represents a clinical conundrum, as these patients have simultaneous increased risk of stroke as well as bleeding complications.^8^, ^9^, ^10^, ^11^ Additionally, all of the more widely used direct-acting oral anticoagulants undergo some degree of renal clearance, thus making pharmacokinetics of these agents somewhat unpredictable in the setting of renal dysfunction.^12^^,^^13^ More recently, left atrial appendage occlusion (LAAO) using a Watchman device has emerged as an alternative strategy to oral anticoagulation in mitigating stroke risk in selected patients with AF.^14^, ^15^, ^16^ Unfortunately, the landmark trials comparing the efficacy and safety of LAAO with warfarin have very limited participation of AF patients with concomitant renal failure.^14^^,^^15^ Additionally, few retrospective and largely single-center studies have demonstrated conflicting outcomes after LAAO with Watchman implantation in AF patients with CKD and ESRD.^17^, ^18^, ^19^, ^20^ The purpose of the current study is to assess the association of CKD or ESRD with risk of procedural complications and inpatient adverse events in AF patients implanted with a Watchman device from a large, nationally representative, and contemporary sample of the United States population.

## Methods

### Data source

Data from National Inpatient Sample (NIS) was used for the purpose of our current study. We analyzed the NIS database from years 2015–2018 for Watchman device implantations. The year 2015 was taken as a start year for our study because the Watchman device was approved by the Food and Drug Administration in March of 2015. The NIS is made possible by a Federal-State-Industry partnership sponsored by the Agency for Healthcare Research and Quality. The NIS is derived from nonfederal hospitals in all states and can be used for computing national estimates of healthcare utilization, costs, and outcomes.^21^ The NIS provides discharge weights that are used for estimation of disease and procedure trends nationally. Owing to the de-identified nature of the NIS dataset, the need for informed consent and Institutional Review Board approval is waived. The NIS adheres to the 2013 Declaration of Helsinki for conduction of human research.

### Study population

Watchman device implantations were identified using International Classification of Diseases, 9th Revision, Clinical Modification (ICD-9-CM) and International Classification of Diseases, 10th Revision, Clinical Modification (ICD-10-CM) codes of 37.90 and 02L73DK, respectively, from our dataset. Patients younger than 18 years and those with missing demographic data were excluded. The study sample was stratified on the basis of renal function into 3 groups (no CKD, CKD, and ESRD). CKD patients were identified using ICD-9-CM codes 585.3 and 585.4 and ICD-10-CM codes N18.3 and N18.4. ESRD patients were identified using ICD-9-CM codes 585.5 and 585.6 and ICD-10-CM codes N18.5 and N18.6 (Supplemental Data). Baseline characteristics, procedural complications, and inpatient outcomes including mortality (reported as a distinct categorical variable in the dataset), length of stay, and hospitalization costs were compared in Watchman recipients based on baseline renal function (no CKD, CKD, and ESRD). The prevalence of acute kidney injury (AKI) was also compared between CKD and no-CKD Watchman LAAO recipients. We also analyzed independent association of both CKD and ESRD with outcomes of major complications (defined as composite of pericardial effusion requiring intervention, cardiac arrest, ischemic stroke / transient ischemic attack, hemorrhagic stroke, systemic embolism, myocardial infarction, and peripheral vascular complications, which included arteriovenous fistula, pseudoaneurysm, access site hematoma, retroperitoneal bleeding, and venous thromboembolism), inpatient mortality, prolonged hospital stay (defined as length of stay >1 day), and increased hospitalization cost (median hospitalization cost >$24,663). Additionally, the independent association of CKD with AKI was analyzed. For computing hospitalization costs, the cost-to-charge ratio files supplied by the Healthcare Cost and Utilization Project were applied to the total hospital charges and adjusted for inflation to December 2018.

### Statistical analysis

Descriptive statistics are presented as frequencies with percentages for categorical variables and as median with interquartile range (IQR) for continuous variables. Baseline characteristics were compared using a Pearson χ^2^ test and Fisher exact test for categorical variables and the Kruskal-Wallis H test for continuous variables. For crude comparison of procedural complications and in-hospital outcomes among the study groups, the Pearson χ^2^ test was used. For assessment of the independent association of both CKD and ESRD with outcomes including major complications, inpatient mortality, length of stay >1 day, median hospitalization cost >$24,663, and AKI, a single-step multivariable logistic regression model was used. Age, sex, race/ethnicity, CHA₂DS₂-VASc score, and 29 Elixhauser comorbidities (heart failure, valvular disease, pulmonary circulation disease, peripheral vascular disease, paralysis, neurological disorders, chronic pulmonary disease, diabetes without complications, diabetes with chronic complications, hypothyroidism, hypertension, renal failure, liver disease, peptic ulcer, acquired immune deficiency syndrome, lymphoma, metastatic cancer, solid tumor without metastasis, collagen vascular disease, coagulopathy, obesity, weight loss, fluid and electrolyte disorders, chronic blood loss anemia, deficiency anemia, alcohol abuse, drug abuse, psychoses, and depression) were used for adjustment. A *P* value of <.05 was considered statistically significant. All statistical analyses were performed using SPSS version 26 (IBM Corp, Armonk, NY) and R version 3.6. Because of the complex survey design of the NIS, sample weights, strata, and clusters were applied to raw data to generate national estimates.

## Results

A total of 36,065 patients underwent Watchman implantation in our study after the relevant exclusion criteria were applied. Of these, 31,405 (87.1%) patients had normal kidney function (no CKD group), 3545 (9.8%) patients had CKD, and 1155 (3.1%) patients had ESRD. Baseline characteristics of the study population are shown in Table 1. In the overall cohort, approximately 41.6% of patients were women. The overall prevalence of white, Black, Hispanic, and patients of other race was 86%, 4.2%, 6%, and 3.8%, respectively. Of patients with AF undergoing Watchman LAAO implantation, older patients were more likely to have CKD (median age 77.5 years, IQR 73–83), whereas younger patients were more likely to have ESRD (median age 71 years, IQR 65–78). The prevalence of both CKD (35.5%) and ESRD (32.3%) was lower in female AF patients undergoing Watchman LAAO implantation when compared to no CKD (42.7%). CKD (7.4%) and ESRD (14.5%) were more prevalent in Black Watchman recipients when compared to no CKD (3.5%). CKD and ESRD Watchman recipients had higher burden of key comorbidities such as heart failure (55.9% and 52.5% vs 30.4%, *P* < .01), coronary artery disease (60.1% and 65.5% vs 46.8%, *P* < .01), hypertension (60.0% and 65.0% vs 54.3%, *P* < .01), obesity (22.1% and 19.3% vs 15.3%, *P* < .01), and peripheral vascular disease (11.4% and 10.8% vs 9.7%, *P* < .01).

**Table 1:** Baseline characteristics of the study population of atrial fibrillation patients undergoing Watchman implantations stratified based on renal function category

Variable	No CKD (n = 31,405)	CKD (n = 3545)	ESRD (n = 1115)	*P* value
Age, median (IQR) years	77 (71–82)	77.5 (73–83)	71 (65–78)	<.01
Female	13,400 (42.7)	1260 (35.5)	360 (32.3)	<.01
Age				
<65	2295 (7.3)	205 (5.8)	255 (22.9)	<.01
65–74	10,110 (32.2)	960 (27.1)	475 (42.6)	
≥75	19,000 (60.5)	2380 (67.1)	385 (34.5)	
Race				
White	26,405 (86.8)	2920 (85.3)	750 (68.2)	<.01
Black	1065 (3.5)	255 (7.4)	160 (14.5)	
Hispanic	1815 (6.0)	130 (3.8)	145 (13.2)	
Asian or Pacific Islander	510 (1.7)	50 (1.5)	25 (2.3)	
Native American	140 (0.5)	0 (0.0)	<10 (<1)	
CHA₂DS₂-VASc score				
0	115 (0.4)	<10 (<0.3)	<10 (<1)	<.01
1	1000 (3.2)	75 (2.1)	80 (7.2)	
2	4520 (14.4)	350 (9.9)	225 (20.2)	
3	9750 (31.0)	1005 (28.3)	370 (33.2)	
4	9520 (30.3)	1220 (34.4)	270 (24.2)	
5	4665 (14.9)	665 (18.8)	110 (9.9)	
≥6	1835 (5.8)	220 (6.2)	55 (4.9)	
Median score	4 (3–4)	4 (3–4)	3 (2–4)	
Comorbidities				
Deficiency anemia	955 (3.0)	190 (5.4)	25 (2.2)	<.01
Congestive heart failure	9535 (30.4)	1980 (55.9)	585 (52.5)	<.01
Chronic pulmonary disease	6630 (21.1)	955 (26.9)	310 (27.8)	<.01
Coagulopathy	1250 (4.0)	200 (5.6)	70 (6.3)	<.01
Coronary artery disease	14,690 (46.8)	2130 (60.1)	730 (65.5)	<.01
Diabetes	6735 (21.4)	185 (5.2)	40 (3.6)	<.01
Hypertension	17,060 (54.3)	2127 (60)	724 (65)	<.01
Liver disease	740 (2.4)	135 (3.8)	65 (5.8)	<.01
Obesity	4795 (15.3)	785 (22.1)	215 (19.3)	<.01
Peripheral vascular disorders	3040 (9.7)	405 (11.4)	120 (10.8)	<.01
Valvular disease	1735 (5.5)	340 (9.6)	95 (8.5)	<.01
Weight loss	140 (0.4)	15 (0.4)	<10 (<1)	.98
Hospital location				
Rural	555 (1.8)	70 (2.0)	25 (2.2)	.01
Urban non-teaching	2975 (9.5)	335 (9.5)	70 (6.3)	
Urban teaching	27,875 (88.8)	3140 (88.6)	1020 (91.5)	
Bed size of the hospital				
Small	3355 (10.7)	470 (13.3)	80 (7.2)	<.01
Medium	6790 (21.6)	660 (18.6)	245 (22.0)	
Large	21,260 (67.7)	2415 (68.1)	790 (70.9)	
Census divisions				
New England	880 (2.8)	95 (2.7)	20 (1.8)	<.01
Mid-Atlantic	4080 (13.0)	395 (11.1)	175 (15.7)	
East North Central	4290 (13.7)	695 (19.6)	190 (17.0)	
West North Central	2230 (7.1)	395 (11.1)	60 (5.4)	
South Atlantic	6620 (21.1)	740 (20.9)	280 (25.1)	
East South Central	1670 (5.3)	170 (4.8)	35 (3.1)	
West South Central	3935 (12.5)	335 (9.4)	115 (10.3)	
Mountain	3290 (10.5)	260 (7.3)	85 (7.6)	
Pacific	4410 (14.0)	460 (13)	155 (13.9)	
Payer				
Medicare	27,635 (88.2)	3210 (90.7)	1030 (92.4)	
Medicaid	360 (1.1)	40 (1.1)	30 (2.7)	<.01
Private insurance	2750 (8.8)	225 (6.4)	45 (4.0)	
Self-pay	165 (0.5)	<10 (<0.3)	0 (0.0)	
Other	430 (1.4)	55 (1.5)	<10 (<1)	
Median income (quartile)				
0–25th percentile	6250 (20.2)	695 (19.8)	335 (30.2)	<.01
26–50th percentile	7935 (25.7)	945 (26.9)	270 (24.3)	
51–75th percentile	8640 (28.0)	965 (27.5)	335 (30.2)	
76–100th percentile	8080 (26.1)	910 (25.9)	170 (15.3)	

Results are n (%) unless otherwise specified.

For n < 10, the numbers are not reported, as per Healthcare Cost and Utilization Project recommendations.

CKD = chronic kidney disease; ESRD = end-stage renal disease.

**Figure 1:**
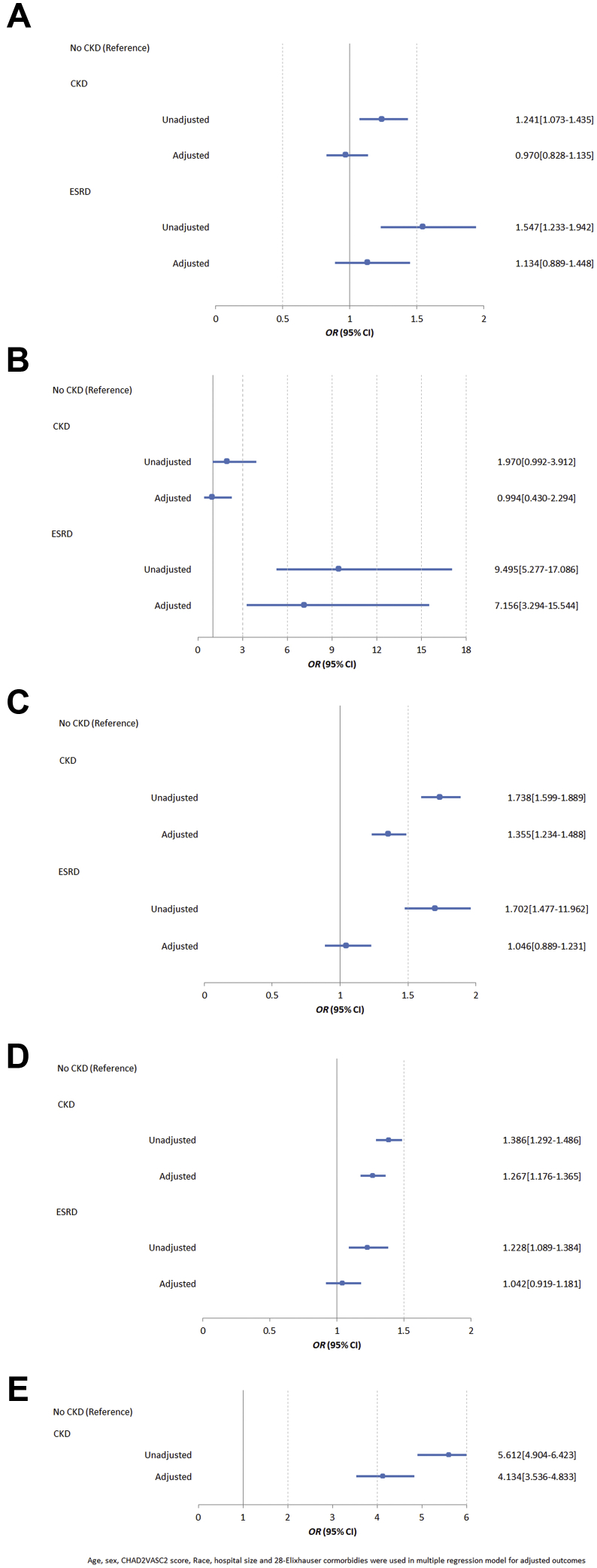
Unadjusted and multivariable adjusted association of both chronic kidney disease (CKD) and end-stage renal disease (ESRD) (reference group no chronic kidney disease) with procedural outcomes including **A:** major complications; **B:** mortality; **C:** prolonged length of stay; **D:** hospitalization cost > median $24,663; and **E:** acute kidney injury.

Crude Watchman procedure-related complications stratified based on kidney function category are shown in Table 2. The prevalence of major complications was higher in AF patients undergoing Watchman LAAO implantation with underlying CKD when compared to patients with no CKD (6.2% vs 5.1%, *P* < .01). The prevalence of pericardial effusion requiring intervention was higher in patients undergoing Watchman LAAO implantation with underlying CKD when compared to patients with no CKD (3.7% vs 2.7%, *P* < .01). The prevalence of any pulmonary complication was also higher in AF patients with CKD undergoing Watchman LAAO implantation compared to patients with no CKD (5.1% vs 2.4%, *P* < .01). Inpatient outcomes after Watchman implantation stratified on the basis of kidney function category are shown in Table 3. CKD was associated with a higher prevalence of crude in-patient mortality (0.3% vs 0.1%, *P* < .01) compared to no CKD. To analyze the independent association of renal function category with adverse outcomes, multivariable logistic regression models were created by adjusting for potential confounders and are shown in Figure 1. CKD was found to be independently associated with prolonged length of stay >1 day (adjusted odds ratio [aOR] 1.355, 95% confidence interval [CI] 1.234–1.488), increased cost of hospitalization above median cost of $24,663 (aOR 1.267, 95% CI 1.176–1.365), and AKI (aOR 4.134, 95% CI 3.536–4.833) in AF patients undergoing Watchman LAAO implantation.

**Table 2:** Complications in atrial fibrillation patients undergoing Watchman implantation stratified based on renal function category

Variables	No CKD (n = 31,405)	CKD (n = 3545)	ESRD (n = 1115)	*P* value
Overall complications	3050 (9.7)	505 (14.2)	195 (17.5)	<.01
Major complications†	1590 (5.1)	220 (6.2)	85 (7.6)	<.01
Any cardiovascular complication	935 (3.0)	140 (3.9)	45 (4.0)	<.01
Percutaneous coronary intervention	80 (0.3)	0 (0.0)	0 (0.0)	<.01
Cardiac arrest	45 (0.1)	20 (0.6)	<10 (<1)	<.01
Heart block	295 (0.9)	40 (1.1)	<10 (<1)	.12
ST-elevation myocardial infarction	25 (0.1)	<10 (<0.3)	0 (0.0)	<.01
Non-ST-elevation myocardial infarction	90 (0.3)	15 (0.4)	<10 (<1)	<.01
Pericardial effusion requiring intervention	855 (2.7)	130 (3.7)	45 (4.0)	<.01
Pericarditis	80 (0.3)	15 (0.4)	<10 (<1)	.11
Cardiogenic shock	80 (0.3)	<10 (<0.3)	<10 (<1)	<.01
Any systemic complication	45 (0.2)	<10 (<0.3)	0 (0.0)	.4
Anaphylaxis	<10 (<0.1)	<10 (<0.3)	0 (0.0)	.01
Arterial thrombosis	30 (0.1)	0 (0.0)	0 (0.0)	.11
Septic shock	<10 (<0.1)	0 (0.0)	0 (0.0)	.48
Any peripheral vascular complication	400 (1.3)	50 (1.4)	20 (1.8)	.27
Arteriovenous fistula	80 (0.3)	15 (0.4)	<10 (<1)	.12
Pseudoaneurysm	95 (0.3)	<10 (<0.3)	0 (0.0)	.18
Hematoma	115 (0.4)	25 (0.7)	<10 (<1)	.01
Retroperitoneal bleeding	25 (0.1)	<10 (<0.3)	0 (0.0)	<.01
Venous thromboembolism	110 (0.4)	0 (0.0)	<10 (<1)	<.01
Any neurological complication	265 (0.8)	20 (0.6)	<10 (<1)	.20
Hemorrhagic stroke	100 (0.3)	0 (0.0)	<10 (<1)	<.01
Ischemic stroke	65 (0.2)	<10 (<0.3)	0 (0.0)	.20
Transient ischemic attack	100 (0.3)	<10 (<0.3)	<10 (<1)	.69
Any gastrointestinal or hematological complication	1325 (4.2)	215 (6.2)	75 (7.2)	.06
Gastrointestinal bleeding	745 (2.4)	100 (2.8)	35 (3.1)	.08
Need for blood transfusion	580 (1.8)	115 (3.2)	40 (3.6)	<.01
Any pulmonary complications	745 (2.4)	180 (5.1)	95 (8.5)	<.01
Respiratory failure	415 (1.3)	115 (3.2)	35 (3.1)	<.01
Pneumothorax	35 (0.1)	<10 (<0.3)	0 (0.0)	.01
Pleural effusion	120 (0.4)	25 (0.7)	25 (2.2)	<.01
Pneumonia	115 (0.4)	25 (0.7)	<10 (<1)	.01
Need for prolonged ventilation (>36 hours)	365 (1.2)	80 (2.3)	60 (5.4)	<.01
Renal complications				
Acute kidney injury	620 (2.0)	360 (10.2)	-	<.01

Results are n (%).

For n < 10, the absolute numbers are not reported, as per Healthcare Cost and Utilization Project recommendations.

CKD = chronic kidney disease; ESRD = end-stage renal disease.

† Composite of pericardial effusion requiring intervention, cardiac arrest, ischemic stroke/ transient ischemic attack, hemorrhagic stroke, systemic embolism, myocardial infarction and peripheral vascular complications which included AV fistula, pseudoaneurysm, access site hematoma, retroperitoneal bleeding and venous thromboembolism.

**Table 3:** Hospital outcomes and resource utilization in atrial fibrillation patients undergoing Watchman implantation stratified based on renal function category

Variables	No CKD (n = 31,405)	CKD (n = 3545)	ESRD (n = 1115)	*P* value
Died at discharge, n (%)	45 (0.1)	<10 (0.3)	15 (1.3)	<.01
Home/routine/self-care, n (%)	28,840 (91.9)	3150 (88.9)	980 (87.9)	<.01
Non-home discharges, n (%)	2540 (8.1)	395 (11.1)	135 (12.1)	
Resource utilization, median (IQR)				
Length of stay, days	1 (1–1)	1 (1–1)	1 (1–1)	<.01
Cost of hospitalization, $	24,345 (18,599–30,409)	25,742 (19,554–31,683)	25,507 (19,637–33,169)	<.01

For n < 10, the absolute numbers are not reported, as per Healthcare Cost and Utilization Project recommendations.

CKD = chronic kidney disease; ESRD = end-stage renal disease.

The crude prevalence of major complications was also higher in AF patients undergoing Watchman LAAO implantation with associated ESRD when compared to no CKD (7.6% vs 5.1%, *P* < .01, Table 2). The prevalence of pericardial effusion requiring intervention was higher in patients undergoing Watchman LAAO implantation with underlying ESRD when compared to patients with no CKD (4% vs 2.7%, *P* < .01). The prevalence of any pulmonary complication was also higher in AF patients with ESRD undergoing Watchman LAAO implantation compared to patients with no CKD (8.5% vs 2.4%, *P* < .01). ESRD was also associated with a higher prevalence of crude in-patient mortality (1.3% vs 0.1%, *P* < .01) compared to no CKD (Table 3). In multivariate adjusted logistic regression analysis, ESRD was found to be independently associated with in-patient mortality (aOR 7.156, 95% CI 3.294–15.544, Figure 1).

## Discussion

In this large and nationally representative sample of AF patients undergoing LAAO with Watchman implantation and associated CKD and ESRD, we report several key findings: (1) The prevalence of renal disease (CKD and ESRD) in contemporary real-world practice of Watchman implantations was approximately 12.6%. (2) CKD and ESRD Watchman recipients had increased prevalence of other comorbidities when compared to patients with no CKD. (3) The crude prevalence of major complications was higher in patients with both underlying CKD and ESRD when compared to patients with no CKD. Furthermore, crude inpatient mortality was also higher in Watchman recipients with CKD and ESRD. (4) CKD was independently associated with prolonged length of stay, increased hospitalization costs, and AKI while ESRD was independently associated with in-patient mortality after Watchman LAAO implantation.

The prevalence of AF in the general population is estimated to be between 0.4% and 1% and is dependent on age, race/ethnicity, and various lifestyle factors.^22^ On the contrary, the prevalence of AF is much higher in patients with renal disease. According to the statistics published by the US Renal Data System (USRDS), about 7% of patients with ESRD on peritoneal dialysis and 13% of patients with ESRD on hemodialysis have concomitant AF.^23^ The prevalence of CKD and ESRD in our cohort of AF patients undergoing LAAO with a Watchman implantation was about 12.6%, which is nearly similar to the statistics reported by USRDS. Few earlier studies have assessed the efficacy and safety of Watchman implantation in AF patients with both CKD and ESRD. Most of these studies were conducted at a single institution with a relatively small sample size and largely conferred conflicting results. In a study of 146 AF (81 CKD and 62 no CKD) patients undergoing LAAO, Brockmeyer and colleagues^17^ demonstrated higher mortality in the CKD group during the follow-up period (10.5/100 person-years vs 4.2/100 person-years). In another study of 300 AF patients undergoing LAAO with a Watchman device (151 CKD and 149 no CKD), Xue and colleagues^18^ showed no difference in the rate of periprocedural complications between CKD and no-CKD cohorts (3.3% vs 3.4%, *P* = 1). Additionally, during the follow-up period of nearly 2 years, all-cause mortality was not statistically different among CKD and no-CKD patients, although there was a trend towards increased mortality in the CKD group (15.2% vs 8.1%, *P* = .07). In a study of 14 AF patients on chronic dialysis, Cruz-González and colleagues^19^ evaluated the long-term efficacy and safety of LAAO using Watchman (7 patients), Amulet (6 patients), and Ultraseal (1 patient) devices. No significant periprocedural complication was noted in their cohort of 14 patients. In addition, no strokes or deaths were reported by their study at the end of the follow-up period. In another study of 92 AF patients on chronic dialysis undergoing LAAO using Watchman (47 patients), Amulet (42 patients), and LAmbre (3 patients) devices, Genovesi and colleagues^20^ demonstrated no difference in bleeding events at 3 months of follow-up between oral anticoagulant–treated and LAAO groups (adjusted HR 1.65, 95% CI 0.43–6.33). They also noted increased mortality in AF patients on oral anticoagulant treatment at maximum follow-up period of 2 years when compared to the LAAO treatment group (adjusted HR 2.76, 95% CI 1.31–5.86). Such improved outcomes witnessed in these single-center studies in AF patients with concomitant renal dysfunction and undergoing LAAO device implantation can be associated with accrual of experience by the implanting physicians, as they were likely underpowered to evaluate in-hospital adverse events from the LAAO procedure itself. In contrast, our large real-world contemporary study of LAAO with a Watchman device had markedly larger numbers of patients and power to detect a difference, and showed increased mortality, especially in ESRD patients, even after adjustment of potential confounding variables (adjusted OR 3.221, 95% CI 1.732–5.99).

The crude prevalence of major complications was higher in our cohort of CKD and ESRD patients after LAAO with a Watchman device compared to no-CKD patients. However, after multivariate adjustment of potential confounding variables, this difference was attenuated. Patients with CKD and ESRD have significant comorbidities, as depicted by earlier studies^6^^,^^23^ and as also witnessed in our current cohort of patients implanted with a Watchman device, that may have contributed to procedural complications in the unadjusted analysis in these subgroups of patients. Additionally, it is plausible that the independent association of CKD with prolonged length of stay and increased hospitalization costs is related to a higher prevalence of AKI in these patients. The development of AKI in CKD patients after LAAO with a Watchman device may have resulted in a need for dialysis, which may prolong the hospital stay and increase the health care costs. Our study also demonstrated ESRD as an independent predictor of mortality in AF patients undergoing LAAO with a Watchman device. Earlier studies have shown increased mortality in ESRD patients with coronary artery disease undergoing percutaneous coronary intervention.^24^^,^^25^ The exact etiology of worsened inpatient mortality in ESRD patients after a Watchman implantation is unclear and may be related to an overall worsened disease state in these patients. Our dataset is limited in analyzing this association, but this subject should be the focus of future investigations.

### Limitations

The results of our study should be interpreted in the context of the following key limitations. First, the NIS relies on ICD codes for disease and procedure identification, which may be subject to errors. It is, however, worth pointing out that NIS has a robust quality control program that minimizes miscoding and ensures data integrity.^21^ Second, the NIS censors outcomes at discharge and patients are not longitudinally followed, and hence long-term outcomes of stroke and bleeding complications after Watchman implantation cannot be ascertained from the dataset. Third, the NIS does not contain information on post-LAAO antiplatelet and anticoagulation strategy, which can be quite variable in CKD and ESRD patients as compared to the general population undergoing a Watchman implant and can affect procedural outcomes in the follow-up period. Fourth, the NIS does not capture information on certain periprocedural variables, such as type and amount of contrast used during the Watchman LAAO implantation and laboratory tests, so quantitative assessment of renal function is not possible. Fifth, the NIS only caters to inpatient admissions and does not provide information on outpatient encounters. However, it should be noted that inpatient admission is often required for reimbursement of an LAAO with a Watchman device,^26^ and hence our study constitutes a well-representative national sample of Watchman implantations in the United States in the contemporary period. Sixth, although we were exhaustive with adjusting for various clinical covariates with respect to our multivariate modeling, residual confounding owing to unknown variables such as social determinants of health cannot be ruled out entirely in our cohort of Watchman LAAO recipients.

## Conclusion

In conclusion, our study of real-word Watchman implantations in the United States showed that the overall prevalence of CKD and ESRD is approximately 13% in such patients. The crude prevalence of major procedural complications was higher in AF patients undergoing Watchman LAAO implantation with associated CKD and ESRD. The crude mortality was also higher in AF patients undergoing Watchman implantation if they have concomitant CKD or ESRD. After multivariate adjustment, CKD was independently associated with prolonged length of stay, increased hospitalization costs, and AKI, while ESRD was independently associated with in-patient mortality. These results have important implications in stratification of such patients and can guide physicians with respect to risk/benefit discussion of LAAO in patients with significant renal disease. Further large-scale clinical studies are required to analyze the long-term outcomes and net clinical benefit of LAAO in AF patients with CKD or ESRD.

## Funding Sources

This research did not receive any specific grant from funding agencies in the public, commercial, or not-for-profit sectors.

## Disclosures

Dr Hsu reports receiving honoraria from Medtronic, Abbott, Boston Scientific, Biotronik, Janssen Pharmaceuticals, Bristol-Myers Squibb, Altathera Pharmaceuticals, Zoll Medical, and Biosense-Webster and research grants from Biotronik and Biosense-Webster, and has equity interest in Acutus Medical and Vektor Medical.

## Authorship

All authors attest they meet the current ICMJE criteria for authorship.

## Patient Consent

Owing to the de-identified nature of the NIS dataset, the need for informed consent is waived.

## Ethics Statement

The NIS adheres to the 2013 Declaration of Helsinki for conduction of human research. Owing to the de-identified nature of the NIS dataset, the need for Institutional Review Board approval is waived.
