# Supplementary material for: Association of chronic kidney disease and end-stage renal disease with procedural complications and in-hospital outcomes from left atrial appendage occlusion device implantation in patients with atrial fibrillation: Insights from the national inpatient sample of 36,065 procedures
Source: Heart Rhythm O2. 2021 Aug 21;2(5):472–9. doi: 10.1016/j.hroo.2021.08.002 (PMC8505197; doi:10.1016/j.hroo.2021.08.002)
Supplement: Supplemental Data [file mmc1.docx]

# ICD-9 and ICD 10 codes

**Watchmen Device:**

3790 and 02L73DK

**CKD**

Chronic kidney disease, Stage III (moderate) convert 585.3 & N18.3

Chronic kidney disease, Stage IV (severe) convert 585.4 & N18.4

**ESRD**

Chronic kidney disease, Stage V convert 585.5 & N18.5

End stage renal disease convert 585.6 & N18.6

1. **Cardiovascular:**
2. Cardiac Arrest/CPR procedure code: 996.0 and 5A12012
3. STEMI: 410 (except 410.7) and I2101,02,03,09,i2111,i219,i212,i2121
4. NSTEMI or type II M: 410.7, I21A,I21A1I21A9,I214
5. Air Embolism: 9580 and T800XXA
6. Heart Failure: 42821,42823,31,33,43 and I5021,23,31,33,41,43
7. Heart Block complete: 426.0 and I442
8. Percutaneous coronary intervention: 3601-09, 0270, 0271,0272,0273
9. Mitral or aortic valve disorder: 4240 and 4241; I34 and I35
10. Pericardial effusion/Hemopericardium: 423.0 and I31.2

10. Cardiac Tamponade: 423.3 and I314

11. Pericarditis: 420.91, 420.91,420.99 and I300,I301,I308,I309

12. Need for Pericardiocentesis: 370 AND 0W9D3, 0W9D4

13. Cardiogenic Shock: 78551, R570, T8111XA

14. Need for diagnostic left heart catheterization:3722, B2100, B2101, B211, B212,B213,B215

**B. Systemic:**

15. Anaphylaxis T78, 9950-9954 & 9956

16. Arterial thrombosis: 444x & I74x

17. Deep venous thrombosis 453x, i82

18. Septic shock: 78552, R65.21

**C. Peripheral Vascular:**

19. AV fistula: 42090,91,99, I300,I308,I309

20. Pseudoaneurysm: 442,I72

21. Local site hematoma: 99812,13,72992,M7981, L763

22. Local site bleeding: 99811, L760,L761,L762

**D. Neurologic:**

23. Hemorrhagic stroke: 430,431,432,I60,I61,I62

24. Ischemic stroke: 433,434 ,436,I63

25. TIA: 435,G45

**E. Bleeding/Hematological complications:**

26. GI bleeding: 578, K92

27. Retroperitoneal Bleeding: 56881, K661

28. Hemothorax after procedure or unknown: 8602, 51189, J942

29. Need for blood products transfusion: 990x, 3023x

**F. Pulmonary:**

30. Post procedure or iatrogenic Pneumothorax and airleak: 5121-2,J93.5

31. Pleural Effusion: 5111,51181,51189,5119, J90,J918

32. Pneumonia bacterial: 481-486; J13-J18

33. Pulmonary embolism: 4151, I26

34. Respiratory failure: 51881,51851,51882,51884, J960,J962,J969

G.

Hemodialysis procedure code:3995, 5A1D70Z, 5A1D80Z, 5A1D90Z, 5A1D00Z, 5A1D60Z

AKI: 584, N17
